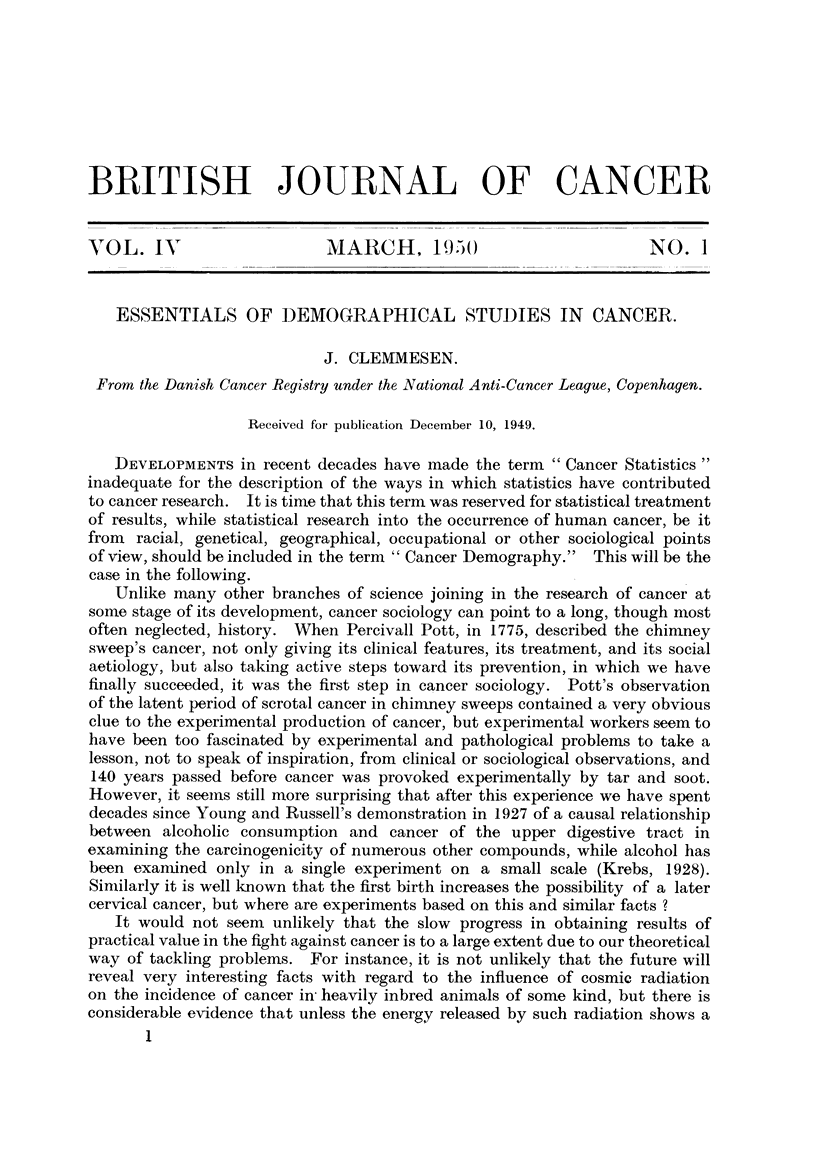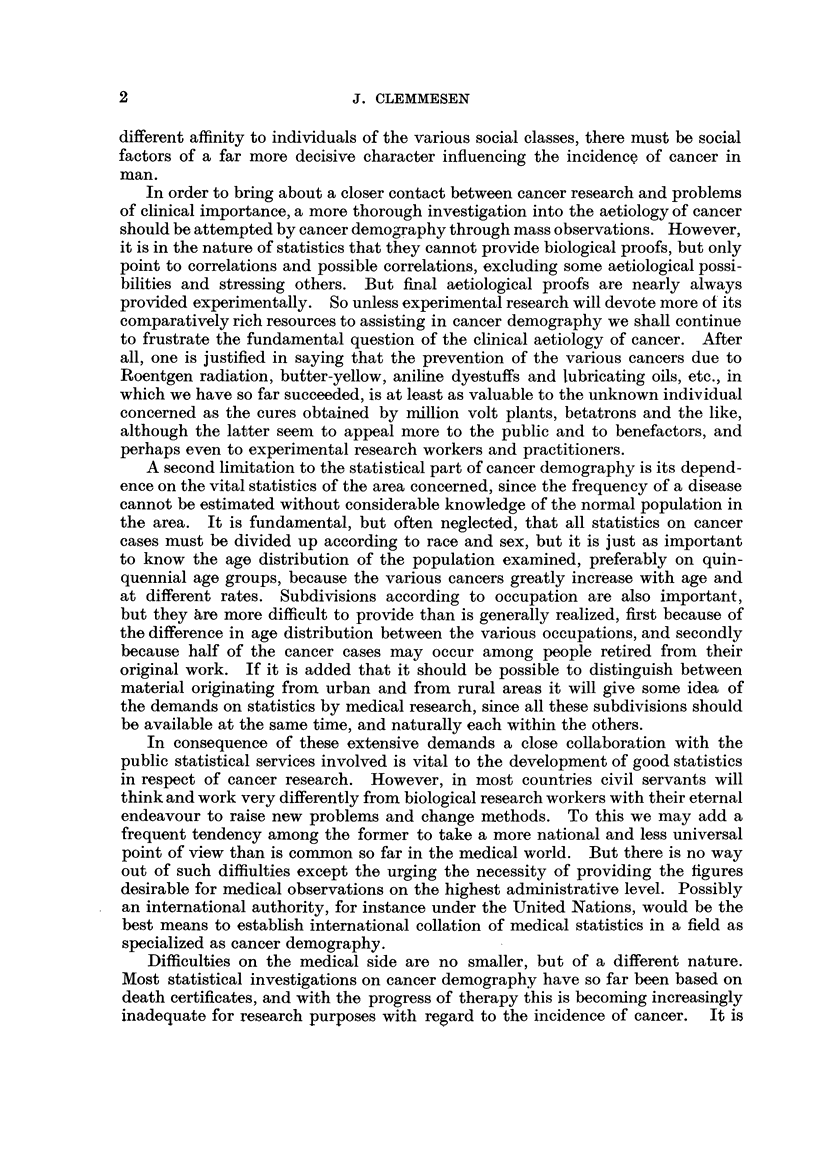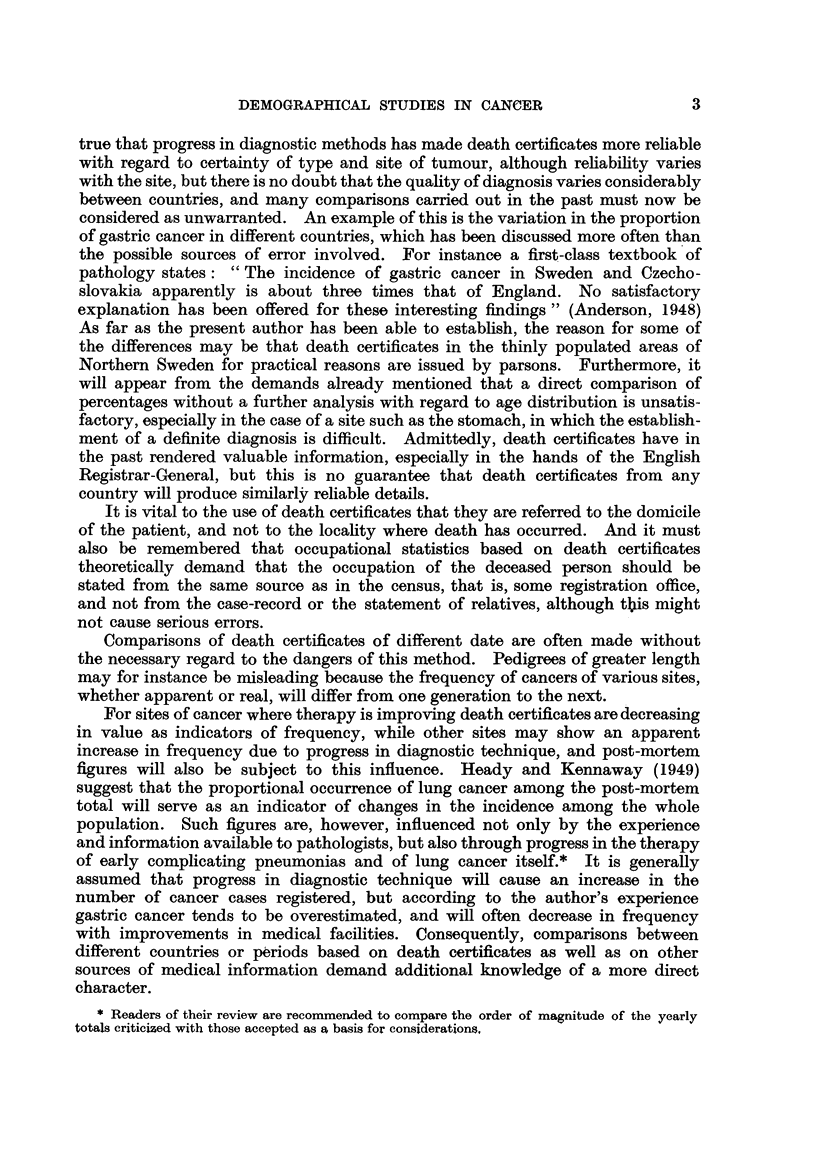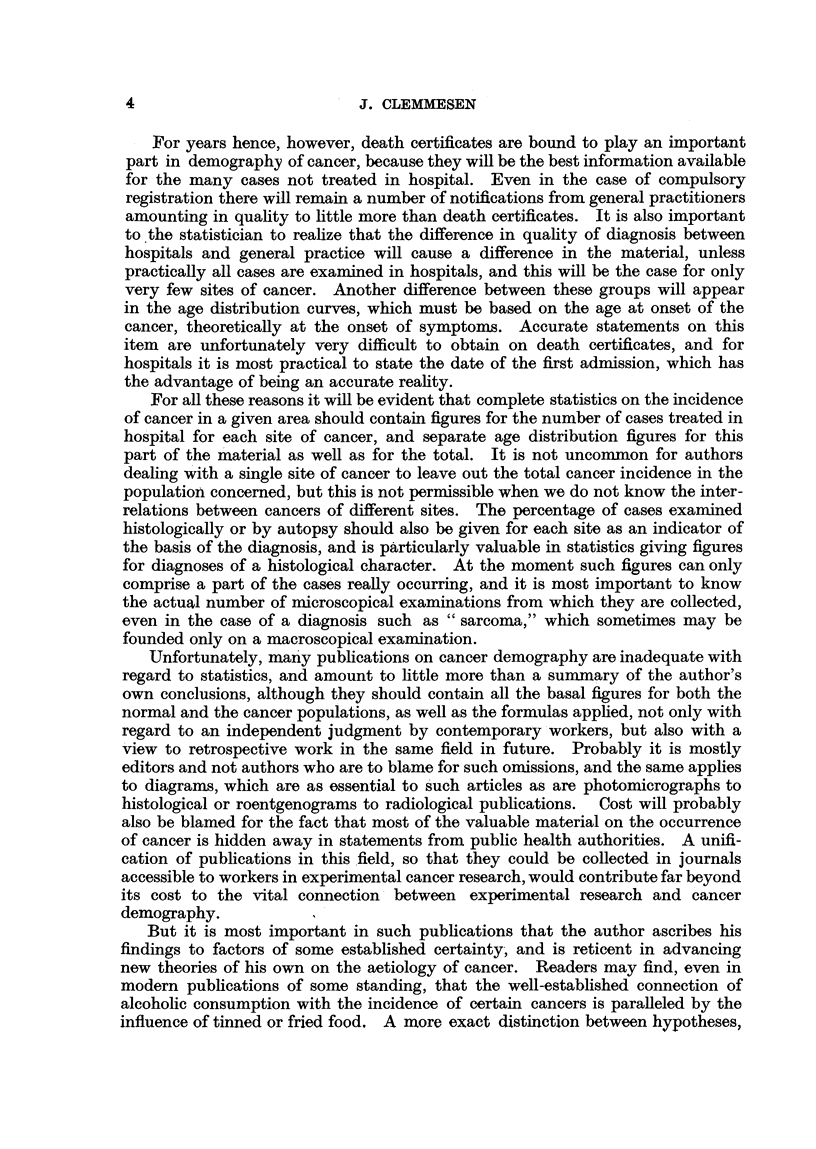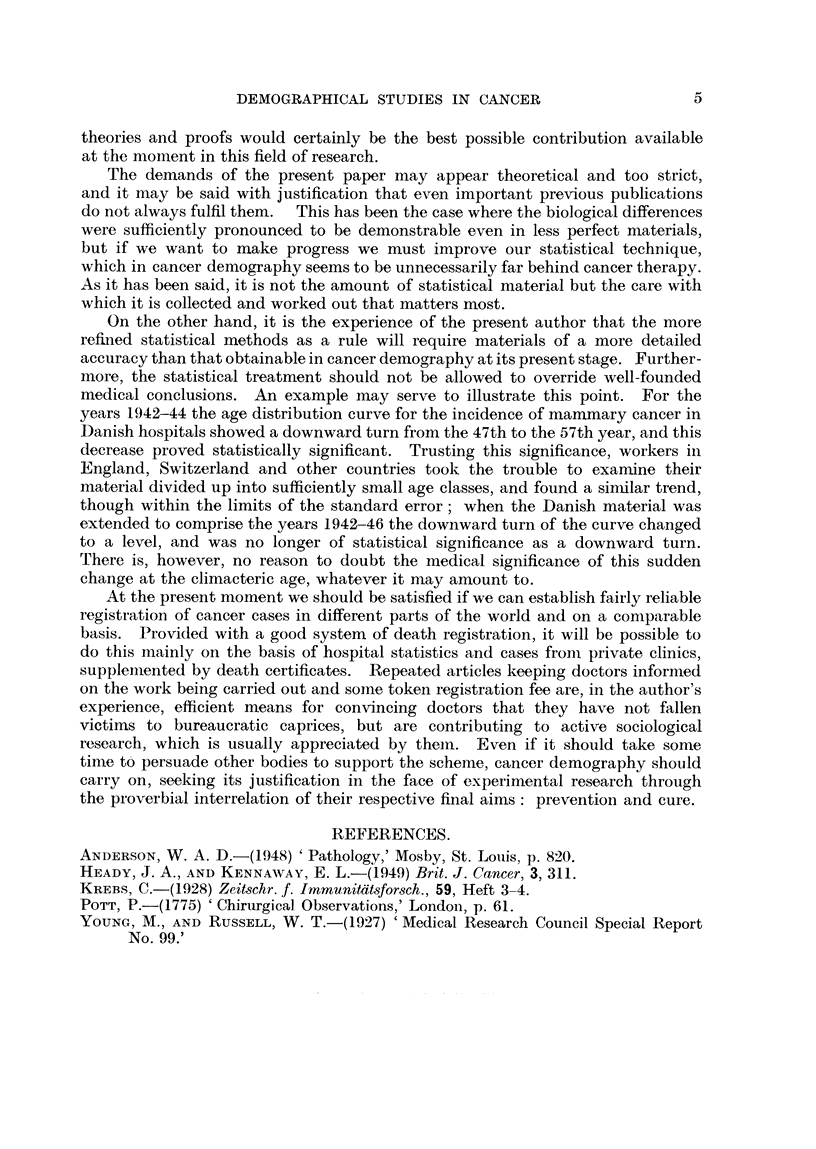# Essentials of Demographical Studies in Cancer

**DOI:** 10.1038/bjc.1950.1

**Published:** 1950-03

**Authors:** J. Clemmesen


// BRITISH JOURNAL OF CANCER

VOL. IV MARCH, 1950 NO. 1

### ESSENTIALS OF DEMOGRAPHICAL STUDIES IN CANCER.

J. CLEMMESEN.

*From the Danish Cancer Registry under the National Anti-Cancer League, Copenhagen.*

Received for publication December 10, 1949.

DEVELOPMENTS in recent decades have made the term "Cancer Statistics" inadequate for the description of the ways in which statistics have contributed to cancer research. It is time that this term was reserved for statistical treatment of results, while statistical research into the occurrence of human cancer, be it from racial, genetical, geographical, occupational or other sociological points of view, should be included in the term "Cancer Demography." This will be the case in the following.

Unlike many other branches of science joining in the research of cancer at some stage of its development, cancer sociology can point to a long, though most often neglected, history. When Percivall Pott, in 1775, described the chimney sweep's cancer, not only giving its clinical features, its treatment, and its social aetiology, but also taking active steps toward its prevention, in which we have finally succeeded, it was the first step in cancer sociology. Pott's observation of the latent period of scrotal cancer in chimney sweeps contained a very obvious clue to the experimental production of cancer, but experimental workers seem to have been too fascinated by experimental and pathological problems to take a lesson, not to speak of inspiration, from clinical or sociological observations, and 140 years passed before cancer was provoked experimentally by tar and soot. However, it seems still more surprising that after this experience we have spent decades since Young and Russell's demonstration in 1927 of a causal relationship between alcoholic consumption and cancer of the upper digestive tract in examining the carcinogenicity of numerous other compounds, while alcohol has been examined only in a single experiment on a small scale (Krebs, 1928). Similarly it is well known that the first birth increases the possibility of a later cervical cancer, but where are experiments based on this and similar facts ?

It would not seem unlikely that the slow progress in obtaining results of practical value in the fight against cancer is to a large extent due to our theoretical way of tackling problems. For instance, it is not unlikely that the future will reveal very interesting facts with regard to the influence of cosmic radiation on the incidence of cancer in heavily inbred animals of some kind, but there is considerable evidence that unless the energy released by such radiation shows a different affinity to individuals of the various social classes, there must be social factors of a far more decisive character influencing the incidence of cancer in man.

In order to bring about a closer contact between cancer research and problems of clinical importance, a more thorough investigation into the aetiology of cancer should be attempted by cancer demography through mass observations. However, it is in the nature of statistics that they cannot provide biological proofs, but only point to correlations and possible correlations, excluding some aetiological possibilities and stressing others. But final aetiological proofs are nearly always provided experimentally. So unless experimental research will devote more of its comparatively rich resources to assisting in cancer demography we shall continue to frustrate the fundamental question of the clinical aetiology of cancer. After all, one is justified in saying that the prevention of the various cancers due to Roentgen radiation, butter-yellow, aniline dyestuffs and lubricating oils, etc., in which we have so far succeeded, is at least as valuable to the unknown individual concerned as the cures obtained by million volt plants, betatrons and the like, although the latter seem to appeal more to the public and to benefactors, and perhaps even to experimental research workers and practitioners.

A second limitation to the statistical part of cancer demography is its dependence on the vital statistics of the area concerned, since the frequency of a disease cannot be estimated without considerable knowledge of the normal population in the area. It is fundamental, but often neglected, that all statistics on cancer cases must be divided up according to race and sex, but it is just as important to know the age distribution of the population examined, preferably on quinquennial age groups, because the various cancers greatly increase with age and at different rates. Subdivisions according to occupation are also important, but they are more difficult to provide than is generally realized, first because of the difference in age distribution between the various occupations, and secondly because half of the cancer cases may occur among people retired from their original work. If it is added that it should be possible to distinguish between material originating from urban and from rural areas it will give some idea of the demands on statistics by medical research, since all these subdivisions should be available at the same time, and naturally each within the others.

In consequence of these extensive demands a close collaboration with the public statistical services involved is vital to the development of good statistics in respect of cancer research. However, in most countries civil servants will think and work very differently from biological research workers with their eternal endeavour to raise new problems and change methods. To this we may add a frequent tendency among the former to take a more national and less universal point of view than is common so far in the medical world. But there is no way out of such diffiulties except the urging the necessity of providing the figures desirable for medical observations on the highest administrative level. Possibly an international authority, for instance under the United Nations, would be the best means to establish international collation of medical statistics in a field as specialized as cancer demography.

Difficulties on the medical side are no smaller, but of a different nature. Most statistical investigations on cancer demography have so far been based on death certificates, and with the progress of therapy this is becoming increasingly inadequate for research purposes with regard to the incidence of cancer. It is true that progress in diagnostic methods has made death certificates more reliable with regard to certainty of type and site of tumour, although reliability varies with the site, but there is no doubt that the quality of diagnosis varies considerably between countries, and many comparisons carried out in the past must now be considered as unwarranted. An example of this is the variation in the proportion of gastric cancer in different countries, which has been discussed more often than the possible sources of error involved. For instance a first-class textbook of pathology states : " The incidence of gastric cancer in Sweden and Czechoslovakia apparently is about three times that of England. No satisfactory explanation has been offered for these interesting findings " (Anderson, 1948) As far as the present author has been able to establish, the reason for some of the differences may be that death certificates in the thinly populated areas of Northern Sweden for practical reasons are issued by parsons. Furthermore, it will appear from the demands already mentioned that a direct comparison of percentages without a further analysis with regard to age distribution is unsatisfactory, especially in the case of a site such as the stomach, in which the establishment of a definite diagnosis is difficult. Admittedly, death certificates have in the past rendered valuable information, especially in the hands of the English Registrar-General, but this is no guarantee that death certificates from any country will produce similarly reliable details.

It is vital to the use of death certificates that they are referred to the domicile of the patient, and not to the locality where death has occurred. And it must also be remembered that occupational statistics based on death certificates theoretically demand that the occupation of the deceased person should be stated from the same source as in the census, that is, some registration office, and not from the case-record or the statement of relatives, although this might not cause serious errors.

Comparisons of death certificates of different date are often made without the necessary regard to the dangers of this method. Pedigrees of greater length may for instance be misleading because the frequency of cancers of various sites, whether apparent or real, will differ from one generation to the next.

For sites of cancer where therapy is improving death certificates are decreasing in value as indicators of frequency, while other sites may show an apparent increase in frequency due to progress in diagnostic technique, and post-mortem figures will also be subject to this influence. Heady and Kennaway (1949) suggest that the proportional occurrence of lung cancer among the post-mortem total will serve as an indicator of changes in the incidence among the whole population. Such figures are, however, influenced not only by the experience and information available to pathologists, but also through progress in the therapy of early complicating pneumonias and of lung cancer itself.* It is generally assumed that progress in diagnostic technique will cause an increase in the number of cancer cases registered, but according to the author's experience gastric cancer tends to be overestimated, and will often decrease in frequency with improvements in medical facilities. Consequently, comparisons between different countries or periods based on death certificates as well as on other sources of medical information demand additional knowledge of a more direct character.

* Readers of their review are recommended to compare the order of magnitude of the yearly totals criticized with those accepted as a basis for considerations.

For years hence, however, death certificates are bound to play an important part in demography of cancer, because they will be the best information available for the many cases not treated in hospital. Even in the case of compulsory registration there will remain a number of notifications from general practitioners amounting in quality to little more than death certificates. It is also important to the statistician to realize that the difference in quality of diagnosis between hospitals and general practice will cause a difference in the material, unless practically all cases are examined in hospitals, and this will be the case for only very few sites of cancer. Another difference between these groups will appear in the age distribution curves, which must be based on the age at onset of the cancer, theoretically at the onset of symptoms. Accurate statements on this item are unfortunately very difficult to obtain on death certificates, and for hospitals it is most practical to state the date of the first admission, which has the advantage of being an accurate reality.

For all these reasons it will be evident that complete statistics on the incidence of cancer in a given area should contain figures for the number of cases treated in hospital for each site of cancer, and separate age distribution figures for this part of the material as well as for the total. It is not uncommon for authors dealing with a single site of cancer to leave out the total cancer incidence in the population concerned, but this is not permissible when we do not know the inter-relations between cancers of different sites. The percentage of cases examined histologically or by autopsy should also be given for each site as an indicator of the basis of the diagnosis, and is particularly valuable in statistics giving figures for diagnoses of a histological character. At the moment such figures can only comprise a part of the cases really occurring, and it is most important to know the actual number of microscopical examinations from which they are collected, even in the case of a diagnosis such as "sarcoma," which sometimes may be founded only on a macroscopical examination.

Unfortunately, many publications on cancer demography are inadequate with regard to statistics, and amount to little more than a summary of the author's own conclusions, although they should contain all the basal figures for both the normal and the cancer populations, as well as the formulas applied, not only with regard to an independent judgment by contemporary workers, but also with a view to retrospective work in the same field in future. Probably it is mostly editors and not authors who are to blame for such omissions, and the same applies to diagrams, which are as essential to such articles as are photomicrographs to histological or roentgenograms to radiological publications. Cost will probably also be blamed for the fact that most of the valuable material on the occurrence of cancer is hidden away in statements from public health authorities. A unification of publications in this field, so that they could be collected in journals accessible to workers in experimental cancer research, would contribute far beyond its cost to the vital connection between experimental research and cancer demography.

But it is most important in such publications that the author ascribes his findings to factors of some established certainty, and is reticent in advancing new theories of his own on the aetiology of cancer. Readers may find, even in modern publications of some standing, that the well-established connection of alcoholic consumption with the incidence of certain cancers is paralleled by the influence of tinned or fried food. A more exact distinction between hypotheses, theories and proofs would certainly be the best possible contribution available at the moment in this field of research.

The demands of the present paper may appear theoretical and too strict, and it may be said with justification that even important previous publications do not always fulfil them. This has been the case where the biological differences were sufficiently pronounced to be demonstrable even in less perfect materials, but if we want to make progress we must improve our statistical technique, which in cancer demography seems to be unnecessarily far behind cancer therapy. As it has been said, it is not the amount of statistical material but the care with which it is collected and worked out that matters most.

On the other hand, it is the experience of the present author that the more refined statistical methods as a rule will require materials of a more detailed accuracy than that obtainable in cancer demography at its present stage. Furthermore, the statistical treatment should not be allowed to override well-founded medical conclusions. An example may serve to illustrate this point. For the years 1942–44 the age distribution curve for the incidence of mammary cancer in Danish hospitals showed a downward turn from the 47th to the 57th year, and this decrease proved statistically significant. Trusting this significance, workers in England, Switzerland and other countries took the trouble to examine their material divided up into sufficiently small age classes, and found a similar trend, though within the limits of the standard error ; when the Danish material was extended to comprise the years 1942–46 the downward turn of the curve changed to a level, and was no longer of statistical significance as a downward turn. There is, however, no reason to doubt the medical significance of this sudden change at the climacteric age, whatever it may amount to.

At the present moment we should be satisfied if we can establish fairly reliable registration of cancer cases in different parts of the world and on a comparable basis. Provided with a good system of death registration, it will be possible to do this mainly on the basis of hospital statistics and cases from private clinics, supplemented by death certificates. Repeated articles keeping doctors informed on the work being carried out and some token registration fee are, in the author's experience, efficient means for convincing doctors that they have not fallen victims to bureaucratic caprices, but are contributing to active sociological research, which is usually appreciated by them. Even if it should take some time to persuade other bodies to support the scheme, cancer demography should carry on, seeking its justification in the face of experimental research through the proverbial interrelation of their respective final aims : prevention and cure.

### REFERENCES.

Anderson, W. A. D.—(1948) 'Pathology,' Mosby, St. Louis, p. 820.

Heady, J. A., and Kennaway, E. L.—(1949) *Brit. J. Cancer*, **3**, 311.

Krebs, C.—(1928) *Zeitschr. f. Immunitätsforsch.*, **59**, Heft 3–4.

Pott, P.—(1775) 'Chirurgical Observations,' London, p. 61.

Young, M., and Russell, W. T.—(1927) 'Medical Research Council Special Report No. 99.'